# Use of methotrexate and TNF inhibitors in patients with rheumatoid arthritis–associated interstitial lung disease: a survey of rheumatologists

**DOI:** 10.1007/s10067-024-07068-2

**Published:** 2024-07-31

**Authors:** Elizabeth Park, Rabia Iqbal, Jon T. Giles, Elana J. Bernstein

**Affiliations:** 1 Division of Rheumatology, Department of Medicine, Columbia University Irving Medical Center, 630 W 168thSt, P&S 3-450, New York, NY 10032 USA; 2 Division of Rheumatology, Department of Medicine, Cedars-Sinai Medical Center, Los Angeles, CA USA

Interstitial lung disease (ILD) is among the most common extra-articular manifestations of rheumatoid arthritis (RA), with symptomatic disease prevalent in approximately 10% of patients [1]. Patients with symptomatic RA-ILD have an increased mortality rate, with a median survival of only 2.6 years from the time of ILD diagnosis [1]. While the incidence of other extra-articular manifestations of RA (e.g., pericarditis, vasculitis) has decreased over time, likely due to aggressive use of disease-modifying antirheumatic drugs (DMARDs) [2], the incidence of RA-ILD has not. Therefore, whether conventional synthetic, biologic, or targeted synthetic DMARDs are effective or harmful for RA-ILD remains an unanswered question [3]. The two most commonly prescribed DMARDs for RA are methotrexate (MTX) and tumor necrosis factor inhibitors (TNFi). MTX has been associated with acute hypersensitivity pneumonitis (HP) [4]. Studies have investigated ILD incidence and flares in MTX and TNFi users, although results were mixed; some studies supported associations, while others did not [5, 6]. Due to these equivocal data, there is hesitancy among rheumatologists to prescribe MTX or TNFi in patients with pre-existing RA-ILD.

We conducted a nationwide survey among adult rheumatologists who were registered American College of Rheumatology (ACR) members, examining practices surrounding MTX and TNFi use in RA-ILD. Of note, this survey was conducted prior to the publication of the 2023 ACR clinical practice guidelines for systemic autoimmune rheumatic disease (SARD)-associated ILD [7].

**Fig. 1:**
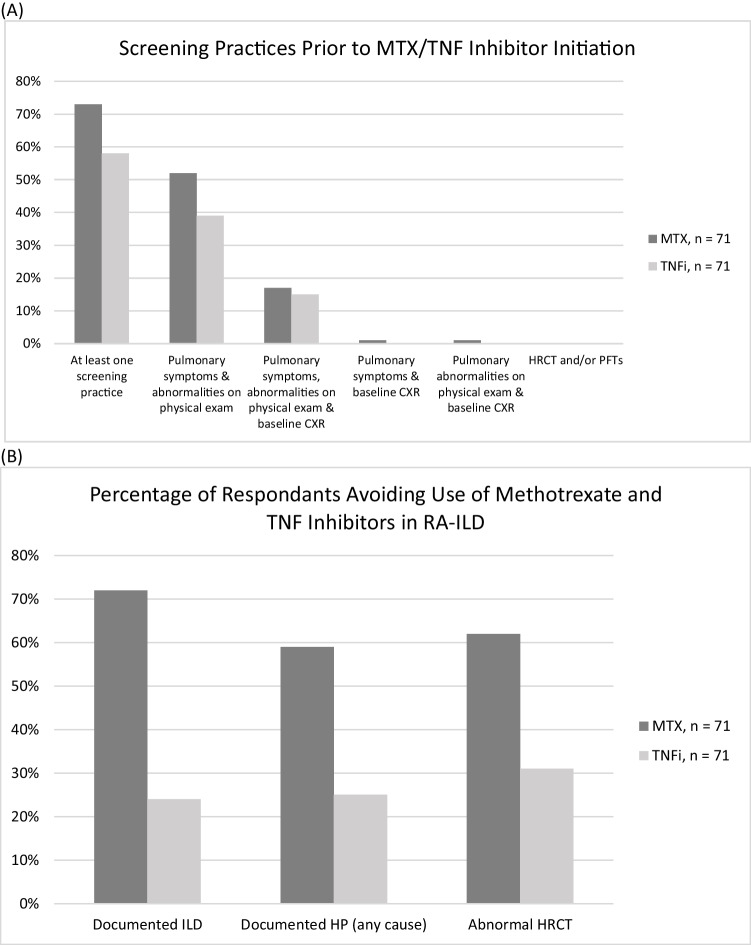
(**A**) Screening practices for RA-ILD prior to methotrexate or TNF inhibitor initiation among responding rheumatologists (**B**) Percent of responding rheumatologists who avoid methotrexate and TNF inhibitors in patients with RA-ILD. CXR = chest X-ray; HP = hypersensitivity pneumonitis; HRCT = high-resolution computed tomography scan of the chest; ILD = interstitial lung disease; MTX = methotrexate; RA-ILD = rheumatoid arthritis-associated interstitial lung disease; TNF = tumor necrosis factor; TNFi =tumor necrosis factor inhibitor

The results of the survey are summarized in Table 1. The survey included multiple-choice and free-text questions. A total of 71 rheumatologists (out of 1227 surveyed; 5.8% response rate) at different levels of training responded to the survey. Approximately 30% of the respondents were fellows, and the majority practiced at a university hospital (65%). Approximately 50% (or fewer) of the respondents stated they would screen with pulmonary symptoms and physical examination abnormalities (Fig. 1A). There were no respondents who stated they would screen with high-resolution computed tomography scan of the chest (HRCT) or pulmonary functions tests (PFTs). Approximately one-third stated they would not perform any screening measures as they did not believe the tests were necessary, due to clinical or cost reasons enumerated in Table 1. Seventy percent indicated they would not repeat tests unless patients developed new or worsening pulmonary symptoms. The majority of respondents would avoid MTX in patients with documented ILD (72%), HP (59%), or abnormal HRCT (62%), while approximately one-quarter of respondents would avoid TNFi in such cases (24% for documented ILD, 25% for HP, 31% for abnormal HRCT) (Fig. 1B). Those who would not avoid MTX/TNFi in ILD cited the fact that there were no robust data to suggest an association between MTX/TNFi and ILD or stated that these medications could treat ILD. Most would not attempt to re-challenge with MTX or TNFi after an episode of HP because many other DMARDs are available. Some responded that they would re-challenge with a different TNFi. In summary, we found that many respondents would perform a combination of pulmonary screening prior to initiation of MTX/TNFi, though most would not repeat screening, and that the majority would avoid MTX in patients with a documented history of ILD or HP.

**Table 1:** Summary of responses

Demographics	*n* = 71	
Trainee status	21 (30%)	
University hospital/medical center	46 (65%)	
Community hospital	7 (10%)	
Private hospital/outpatient clinic	15 (21%)	
Other	6 (8%)	
Screening practices prior to MTX/TNFi initiation	MTX, *n* = 71	TNFi, *n* = 71
At least one screening practice	52 (73%)	58% (41/71)
HRCT and/or PFTs	0 (0%)	0 (0%)
Pulmonary symptoms & abnormalities on physical exam	37 (52%)	28 (39%)
Pulmonary symptoms, abnormalities on physical exam & baseline CXR	12 (17%)	11 (15%)
Pulmonary symptoms & baseline CXR	1 (1%)	0 (0%)
Pulmonary abnormalities on physical exam & baseline CXR	1 (1%)	0 (0%)
Other combinations^a^	1 (1%)	1 (1%)
Reasons for not ordering screening tests	*n* = 25	
Combination of two or more reasons	11 (44%)	
Tests are not necessary	8 (32%)	
Clinical reasons^b^	4 (16%)	
Cost reasons^c^	1 (4%)	
Administrative reasons^d^	0 (0%)	
Repeating screening interval	MTX, *n* = 71	TNFi, *n* = 71
Every 12 months	4 (6%)	4 (6%)
Every 6 months	5 (7%)	2 (3%)
No repeats	50 (70%)	49 (69%)
Other frequency	12 (17%)	12 (17%)
Repeat screening with:	*n*=71	
CXR	7 (10%)	
HRCT	0 (0%)	
PFTs	1 (1%)	
Combination of CXR, HRCT, and/or PFTs	20 (28%)	
Other^e^	5 (7%)	
Avoid the use of MTX/TNFi if:	MTX, *n* = 71	TNFi, *n* = 71
Documented ILD	51 (72%)	17 (24%)
Documented HP (any cause)	42 (59%)	18 (25%)
Abnormal HRCT	44 (62%)	22 (31%)
Re-challenging after acute HP	MTX, *n* = 71	TNFi, *n* = 71
Yes	5 (7%)	12 (17%)
No	64 (90%)	53 (75%)

Methotrexate *(MTX);* Tumor Necrosis Factor inhibitors *(TNFi);* Chest X-Ray *(CXR);* High-Resolution Computed Tomography Scan of the Chest *(HRCT);* Pulmonary Function Tests *(PFTs);* Interstitial Lung Disease *(ILD);* Hypersensitivity Pneumonitis *(HP)*

^a^Other combinations: Quantiferon gold, liver function tests, hepatitis B/C serologies, and history of ETOH abuse

^b^Clinical reasons: Symptoms and/or exams do not suggest clinically significant underlying pulmonary disease

^c^Cost reasons: Tests not covered by health insurance of systems

^d^Administrative reasons: Scheduling CT or PFTs not feasible

^e^Other: Combination of symptoms, exam, 6-minute walk test, CXR, HRCT, and PFTs depending on clinical scenario

The newly released ACR SARD-ILD guidelines will serve as a critical resource to educate and guide clinicians in RA-ILD management. There were some notable differences, including our respondents favoring the use of CXR over HRCT (whereas the guidelines conditionally recommend against CXR as a screening tool for ILD). The majority of respondents would avoid the use of MTX/TNFi in RA-ILD, but the role of these DMARDs in the actual treatment of RA-ILD was not explicitly raised in this survey (while guidelines conditionally recommend against using MTX/TNFi to treat RA-ILD). A lower-than-expected proportion (52% and 39%, respectively, for MTX and TNFi) stated they would screen with pulmonary symptoms/exam abnormalities—both low-cost options and standard care—prior to initiation of DMARDs. Therefore, it may be worthwhile to revisit these practice trends, alongside any potential barriers to implementing the ACR SARD-ILD guidelines, in a follow-up survey.

Our study had some limitations. Selection bias could have occurred as the majority of those who responded were trainees or faculty in academic settings, and those practicing in the community may have been less likely to be included or chosen to participate; thus, the survey may not capture the full, general rheumatologist population, and the results may not be generalizable to rheumatologists who practice outside of academia. Non-responder bias may also have occurred; those who feel strongly about the role of screening tests for ILD may have been more likely to respond to the survey than those with less strong opinions about ILD screening. Nonetheless, despite these potential biases, this survey reveals important practice trends surrounding DMARDs and RA-ILD that mandate further exploration in the advent of the ACR SARD-ILD guidelines.
